# Diethyl 5-amino-2,4,6-triiodo­isophthalate

**DOI:** 10.1107/S1600536809051149

**Published:** 2009-12-04

**Authors:** Jun Wu, Min-Hao Xie, Pei Zou, Ya-Ling Liu, Yong-Jun He

**Affiliations:** a Jiangsu Institute of Nuclear Medicine, Wuxi 214063, People’s Republic of China

## Abstract

The title compound, C_12_H_12_I_3_NO_4_, crystallizes with two mol­ecules in an asymmetric unit. In one of the mol­ecules, the conformation of the O—C—O—C in one ester group is *cis* and *trans* in the other. The corresponding conformations for both the ester groups in the other mol­ecule are *trans*. The I atoms and the benzene rings in the two mol­ecules are approximately coplanar, the I atoms deviating by 0.219 (14), 0.056 (15) and −0.143 (14) Å from the mean plane of the benzene ring in one mol­ecule and 0.189 (14), −0.162 (15) and −0.068 (14) Å in the other. The planes of the ester groups are almost orthogonal to those of the benzene rings in both mol­ecules, forming dihedral angles of 88.1 (4), 72.2 (4), 73.0 (4) and 86.6 (4)°. The mean planes of the benzene rings in the two mol­ecules are inclined at 74.6 (4)° with respect to each other. In the crystal, inter­molecular I⋯O inter­actions [3.138 (7) and 3.144 (7) Å] link the mol­ecules into infinite chains along the *a* axis. In addition, non-classical C—H⋯O hydrogen bonds are observed.

## Related literature

For iodine-based compounds as contrast agents for X-ray imaging, see: Stacul, (2001 ▶); Yu & Watson (1999 ▶); Tonnessen *et al.* (1996 ▶). For a related structure, see: Beck & Sheldrick (2008 ▶).
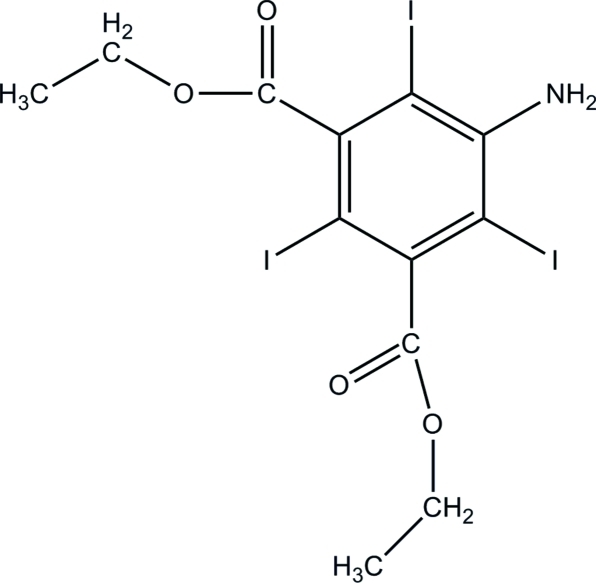


## Experimental

### 

#### Crystal data

C_12_H_12_I_3_NO_4_
*M* *_r_* = 614.93
Monoclinic, 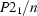

*a* = 9.7410 (8) Å
*b* = 9.6870 (7) Å
*c* = 37.7290 (15) Å
β = 94.430 (3)°
*V* = 3549.5 (4) Å^3^
*Z* = 8
Mo *K*α radiation
μ = 5.29 mm^−1^
*T* = 296 K
0.26 × 0.18 × 0.12 mm

#### Data collection

Enraf–Nonius CAD-4 diffractometer
Absorption correction: ψ scan (*CAD-4 Software*; Enraf–Nonius, 1989 ▶) *T*_min_ = 0.362, *T*_max_ = 0.528
6676 measured reflections
6281 independent reflections
4259 reflections with *I* > 2σ(*I*)
*R*_int_ = 0.045
3 standard reflections every 200 reflections intensity decay: 1%

#### Refinement

*R*[*F* ^2^ > 2σ(*F* ^2^)] = 0.060
*wR*(*F* ^2^) = 0.162
*S* = 1.06
6222 reflections
365 parameters
84 restraints
H-atom parameters constrained
Δρ_max_ = 0.84 e Å^−3^
Δρ_min_ = −1.05 e Å^−3^

### 

Data collection: *CAD-4 Software* (Enraf–Nonius, 1989 ▶); cell refinement: *CAD-4 Software*; data reduction: *XCAD4* (Harms & Wocadlo, 1995 ▶); program(s) used to solve structure: *SHELXS97* (Sheldrick, 2008 ▶); program(s) used to refine structure: *SHELXL97* (Sheldrick, 2008 ▶); molecular graphics: *SHELXTL* (Sheldrick, 2008 ▶); software used to prepare material for publication: *SHELXTL*.

## Supplementary Material

Crystal structure: contains datablocks I, global. DOI: 10.1107/S1600536809051149/pv2229sup1.cif

Structure factors: contains datablocks I. DOI: 10.1107/S1600536809051149/pv2229Isup2.hkl

Additional supplementary materials:  crystallographic information; 3D view; checkCIF report

## Figures and Tables

**Table 1:** Hydrogen-bond geometry (Å, °)

*D*—H⋯*A*	*D*—H	H⋯*A*	*D*⋯*A*	*D*—H⋯*A*
C2*A*—H2*A*2⋯O2*A*^i^	0.97	2.54	3.411 (8)	150
C12*A*—H12*A*⋯O2*B*^ii^	0.96	2.60	3.545 (11)	169

Symmetry codes: (i) 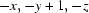; (ii) 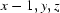.

**Fig. 1.**
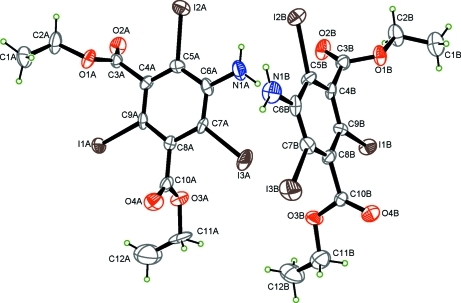
A view of the two molecules of the title compound with the atomic numbering scheme. Displacement ellipsoids were drawn at the 50% probability level.

**Fig. 2.**
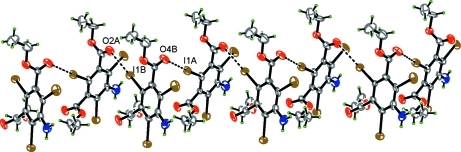
A view of the molecular structure of the title compound showing infinite one dimensional chains formed by I···O interactions (dashed lines).

## supplementary crystallographic information

### Comment

Iodine-based compounds have been in the focus as contrast agents for X-ray imaging (Stacul, 2001). The 1,3,5-triiodobenzene core has been the basis of many contrast agents (Yu & Watson, 1999). The ionic monomer, diatrizoate was one of the first *X*-ray contrast agents in clinical use based on triiodinated benzene (Tonnessen *et al.*, 1996). In this paper, we present the crystal structure of the title compound, (I).

The asymmetric unit of the title compound (Fig. 1) contains two crystallographically independent molecules (A, B) in an asymmetric unit. The three I atoms deviate from the mean-planes of the the phenyl rings, respectively, by 0.219 (14), 0.056 (15) and -0.143 (14) Å for molecule A and 0.189 (14), -0.162 (15) and -0.068 (14) Å for molecule B. Bond lengths and angles are comparable to those observed in a related structure (Beck & Sheldrick, 2008). In molecule A, the conformation of the O–C–O–C is *cis* with respect to the O1A—C3A bond (torsion angle, -90.6 (8)°) and *trans* with respect to the O3A—C11A bond (torsion angle, 158.0 (8)°). On the other hand, the corresponding bonds exhibit *trans* conformations for both the ester groups with torsion angles about O1B—C3B and O3B—C11B bonds being 157.5 (8) and 177.5 (6) °, respectively. The planes of the ester groups in both molecule are almost orthogonal to the benzene rings, as indicated by the dihedral angles of 88.1 (4)° (O1A/O2A/C3A/C4A; C4A—C9A), 72.2 (4)° (O3A/O4A/C8A/C10A; C4A—C9A), 73.0 (4)° (O1B/O2B/C3B/C4B; C4B—C9B) and 86.6 (4)° (O3B/O4B/C8B/C10B/C11B/C12B; C4B—C9B). The dihedral angle between the ring (C4A—C9A) and the ring (C4B—C9B) is 74.6 (4)°.

In the crystal structure, intermolecular I···O interactions of the order 3.138 (7) and 3.144 (7) Å link the molecules into infinite one-dimensional chains along the *a* axis (Fig. 2). In addition, non-classical C—H···O hydrogen bonds are observed (Table 1).

### Experimental

A mixture of 5-amino-2,4,6-triiodoisophthaloyl dichloride (5.95 g, 10 mmol) and ethanol (30 ml) was heated under reflux for four hours to produce diethyl 5-amino-2,4,6-triiodoisophthalate. It was recrystallized from an ethanol solution by slowly evaporating the solvent to obtain crystals suitable for X-ray single-crystal diffraction.

### Refinement

All H atoms were initially located from a difference Fourier map and then were regenerated at ideal positions and treated as riding, with N—H = 0.86 Å, C—H = 0.96–0.97 Å and *U*_iso_(H) = 1.2*U*_eq_ (C, N). The residual electron density was located close to the iodine atoms and was essentially meaningless.  Because the completeness of the data is a bit low, the high angle is restrained at 50.02; and as a result of that, the number of reflections used in refinement is only 6222.

### Crystal data

**Table:**

C_12_H_12_I_3_NO_4_	*F*(000) = 2256
*M**_r_* = 614.93	*D*_x_ = 2.301 Mg m^−^^3^
Monoclinic, *P*2_1_/*n*	Mo *K*α radiation, λ = 0.71073 Å
Hall symbol: -P 2yn	Cell parameters from 5986 reflections
*a* = 9.7410 (8) Å	θ = 3.2–25.2°
*b* = 9.6870 (7) Å	µ = 5.29 mm^−^^1^
*c* = 37.7290 (15) Å	*T* = 296 K
β = 94.430 (3)°	Chunk, colorless
*V* = 3549.5 (4) Å^3^	0.26 × 0.18 × 0.12 mm
*Z* = 8	

### Data collection

**Table:**

Enraf–Nonius CAD-4 diffractometer	4259 reflections with *I* > 2σ(*I*)
Radiation source: fine-focus sealed tube	*R*_int_ = 0.045
graphite	θ_max_ = 25.0°, θ_min_ = 1.1°
ω/2θ scans	*h* = −11→11
Absorption correction: ψ scan (*CAD-4 Software*; Enraf–Nonius, 1989)	*k* = 0→11
*T*_min_ = 0.362, *T*_max_ = 0.528	*l* = 0→43
6676 measured reflections	3 standard reflections every 200 reflections
6281 independent reflections	intensity decay: 1%

### Refinement

**Table:**

Refinement on *F*^2^	Primary atom site location: structure-invariant direct methods
Least-squares matrix: full	Secondary atom site location: difference Fourier map
*R*[*F*^2^ > 2σ(*F*^2^)] = 0.060	Hydrogen site location: inferred from neighbouring sites
*wR*(*F*^2^) = 0.162	H-atom parameters constrained
*S* = 1.06	*w* = 1/[σ^2^(*F*_o_^2^) + (0.085*P*)^2^ + 12.*P*] where *P* = (*F*_o_^2^ + 2*F*_c_^2^)/3
6222 reflections	(Δ/σ)_max_ = 0.004
365 parameters	Δρ_max_ = 0.84 e Å^−^^3^
84 restraints	Δρ_min_ = −1.05 e Å^−^^3^

### Special details

**Table:**

Geometry. All e.s.d.'s (except the e.s.d. in the dihedral angle between two l.s. planes) are estimated using the full covariance matrix. The cell e.s.d.'s are taken into account individually in the estimation of e.s.d.'s in distances, angles and torsion angles; correlations between e.s.d.'s in cell parameters are only used when they are defined by crystal symmetry. An approximate (isotropic) treatment of cell e.s.d.'s is used for estimating e.s.d.'s involving l.s. planes.
Refinement. Refinement of *F*^2^ against ALL reflections. The weighted *R*-factor *wR* and goodness of fit *S* are based on *F*^2^, conventional *R*-factors *R* are based on *F*, with *F* set to zero for negative *F*^2^. The threshold expression of *F*^2^ > σ(*F*^2^) is used only for calculating *R*-factors(gt) *etc*. and is not relevant to the choice of reflections for refinement. *R*-factors based on *F*^2^ are statistically about twice as large as those based on *F*, and *R*- factors based on ALL data will be even larger.

### Fractional atomic coordinates and isotropic or equivalent isotropic displacement parameters (Å^2^)

**Table:**

	*x*	*y*	*z*	*U*_iso_*/*U*_eq_	
I1B	0.91907 (6)	0.24382 (7)	0.092312 (16)	0.04395 (16)	
I1A	0.42314 (6)	0.32088 (7)	0.091767 (17)	0.04606 (17)	
I3A	0.32374 (7)	0.63629 (7)	0.228910 (16)	0.04879 (18)	
I2B	0.81984 (6)	−0.08251 (7)	0.227645 (16)	0.04797 (18)	
I3B	0.50611 (7)	−0.22049 (8)	0.08379 (2)	0.0610 (2)	
I2A	0.00810 (7)	0.78328 (8)	0.08607 (2)	0.0644 (2)	
C7A	0.2827 (8)	0.6055 (9)	0.1725 (2)	0.031 (2)	
O1A	0.2558 (6)	0.5972 (7)	0.03825 (7)	0.0469 (17)	
O3B	0.7559 (6)	−0.0349 (7)	0.03756 (7)	0.0504 (18)	
O1B	0.8887 (4)	0.2728 (4)	0.18292 (16)	0.0426 (17)	
O4A	0.5601 (6)	0.4411 (6)	0.18705 (18)	0.0473 (18)	
O3A	0.3932 (4)	0.2852 (4)	0.18200 (16)	0.0433 (17)	
C3A	0.1782 (9)	0.5254 (10)	0.0593 (2)	0.042 (2)	
O2B	1.0594 (6)	0.1144 (7)	0.18664 (19)	0.0493 (18)	
O4B	0.5915 (7)	0.1160 (8)	0.04744 (18)	0.056 (2)	
C5B	0.7797 (8)	−0.0506 (9)	0.1708 (2)	0.033 (2)	
C10B	0.6797 (8)	0.0387 (9)	0.0585 (2)	0.031 (2)	
O2A	0.0891 (6)	0.4496 (7)	0.04842 (18)	0.0550 (19)	
C6A	0.1885 (8)	0.6946 (9)	0.1533 (2)	0.036 (2)	
C5A	0.1544 (9)	0.6638 (9)	0.1165 (2)	0.036 (2)	
C9B	0.8118 (7)	0.0883 (8)	0.11840 (19)	0.0262 (18)	
N1B	0.6258 (8)	−0.2450 (8)	0.1678 (2)	0.050 (2)	
H1B1	0.6454	−0.2589	0.1902	0.060*	
H1B2	0.5685	−0.2985	0.1560	0.060*	
C9A	0.3143 (8)	0.4725 (8)	0.1186 (2)	0.033 (2)	
N1A	0.1263 (9)	0.8035 (8)	0.1696 (2)	0.056 (2)	
H1A1	0.1446	0.8184	0.1919	0.068*	
H1A2	0.0695	0.8562	0.1574	0.068*	
C4A	0.2167 (7)	0.5527 (8)	0.0986 (2)	0.0280 (19)	
C10A	0.4476 (8)	0.4076 (9)	0.1766 (2)	0.033 (2)	
C4B	0.8418 (8)	0.0606 (8)	0.1547 (2)	0.030 (2)	
C7B	0.6511 (8)	−0.1024 (9)	0.1146 (2)	0.037 (2)	
C6B	0.6866 (8)	−0.1361 (8)	0.1508 (3)	0.038 (2)	
C8A	0.3445 (7)	0.4994 (9)	0.1557 (2)	0.031 (2)	
C12A	0.4169 (10)	0.0442 (10)	0.1878 (3)	0.055 (3)	
H12A	0.3197	0.0535	0.1898	0.066*	
H12B	0.4525	−0.0283	0.2032	0.066*	
H12C	0.4331	0.0219	0.1637	0.066*	
C3B	0.9446 (9)	0.1487 (9)	0.1769 (2)	0.036 (2)	
C8B	0.7160 (8)	0.0048 (9)	0.0982 (2)	0.034 (2)	
C2A	0.2210 (5)	0.5890 (6)	−0.00099 (8)	0.066 (3)	
H2A1	0.2434	0.6767	−0.0115	0.079*	
H2A2	0.1224	0.5756	−0.0053	0.079*	
C11A	0.4865 (7)	0.1753 (7)	0.1980 (3)	0.059 (3)	
H11A	0.5762	0.1802	0.1885	0.071*	
H11B	0.4980	0.1847	0.2236	0.071*	
C1A	0.2940 (11)	0.4747 (10)	−0.0198 (3)	0.079 (6)	
H1A3	0.3820	0.5074	−0.0261	0.095*	
H1A4	0.2392	0.4482	−0.0410	0.095*	
H1A5	0.3068	0.3964	−0.0043	0.095*	
C11B	0.7246 (4)	−0.0172 (6)	−0.00162 (7)	0.075 (4)	
H11C	0.7324	0.0796	−0.0077	0.090*	
H11D	0.6306	−0.0460	−0.0081	0.090*	
C2B	0.9858 (5)	0.3800 (3)	0.1986 (2)	0.055 (3)	
H2B1	1.0744	0.3733	0.1886	0.066*	
H2B2	0.9993	0.3697	0.2242	0.066*	
C12B	0.82239 (15)	−0.1014 (4)	−0.02254 (14)	0.091 (4)	
H12D	0.9144	−0.0669	−0.0180	0.109*	
H12E	0.7952	−0.0942	−0.0475	0.109*	
H12F	0.8192	−0.1964	−0.0154	0.109*	
C1B	0.9166 (8)	0.5171 (5)	0.1888 (3)	0.065 (3)	
H1B3	0.9270	0.5371	0.1642	0.078*	
H1B4	0.9587	0.5891	0.2034	0.078*	
H1B5	0.8205	0.5115	0.1926	0.078*	

### Atomic displacement parameters (Å^2^)

**Table:**

	*U*^11^	*U*^22^	*U*^33^	*U*^12^	*U*^13^	*U*^23^
I1B	0.0448 (3)	0.0435 (4)	0.0429 (3)	−0.0117 (3)	−0.0009 (3)	0.0084 (3)
I1A	0.0493 (4)	0.0430 (4)	0.0450 (3)	0.0190 (3)	−0.0021 (3)	−0.0076 (3)
I3A	0.0498 (4)	0.0544 (4)	0.0417 (3)	−0.0005 (3)	0.0010 (3)	−0.0106 (3)
I2B	0.0499 (4)	0.0517 (4)	0.0420 (3)	0.0022 (3)	0.0012 (3)	0.0126 (3)
I3B	0.0494 (4)	0.0581 (5)	0.0734 (5)	−0.0217 (3)	−0.0083 (3)	−0.0128 (4)
I2A	0.0537 (4)	0.0603 (5)	0.0769 (5)	0.0315 (3)	−0.0088 (4)	0.0136 (4)
C7A	0.025 (4)	0.036 (5)	0.033 (4)	−0.012 (4)	−0.002 (3)	−0.003 (4)
O1A	0.042 (3)	0.059 (4)	0.038 (3)	−0.001 (3)	−0.003 (3)	0.011 (3)
O3B	0.047 (3)	0.066 (4)	0.036 (3)	0.023 (3)	−0.006 (3)	−0.013 (3)
O1B	0.028 (3)	0.027 (3)	0.072 (4)	−0.001 (3)	−0.003 (3)	−0.007 (3)
O4A	0.030 (3)	0.044 (4)	0.065 (4)	−0.007 (3)	−0.010 (3)	0.003 (3)
O3A	0.033 (3)	0.036 (4)	0.060 (4)	0.007 (3)	−0.002 (3)	0.018 (3)
C3A	0.037 (5)	0.036 (5)	0.050 (6)	0.012 (4)	−0.012 (4)	0.004 (4)
O2B	0.024 (3)	0.044 (4)	0.078 (5)	0.002 (3)	−0.014 (3)	0.007 (4)
O4B	0.057 (4)	0.060 (4)	0.048 (4)	0.012 (4)	−0.018 (3)	−0.004 (4)
C5B	0.031 (4)	0.036 (5)	0.030 (4)	−0.001 (4)	−0.005 (3)	0.010 (4)
C10B	0.027 (4)	0.033 (5)	0.034 (4)	0.007 (4)	−0.002 (3)	−0.002 (4)
O2A	0.046 (4)	0.054 (4)	0.061 (4)	−0.018 (3)	−0.014 (3)	0.002 (4)
C6A	0.022 (4)	0.030 (5)	0.056 (6)	0.006 (4)	0.004 (4)	−0.013 (4)
C5A	0.037 (5)	0.028 (4)	0.042 (5)	0.014 (4)	−0.001 (4)	0.012 (4)
C9B	0.020 (4)	0.035 (5)	0.024 (4)	−0.002 (3)	0.004 (3)	−0.004 (3)
N1B	0.046 (5)	0.037 (4)	0.069 (5)	−0.018 (4)	0.011 (4)	0.006 (4)
C9A	0.026 (4)	0.018 (4)	0.056 (5)	0.008 (3)	0.000 (4)	0.000 (4)
N1A	0.057 (5)	0.041 (5)	0.071 (6)	0.011 (4)	0.003 (4)	−0.014 (4)
C4A	0.018 (4)	0.029 (4)	0.037 (4)	−0.001 (3)	−0.001 (3)	0.004 (4)
C10A	0.029 (4)	0.027 (5)	0.040 (5)	0.006 (4)	−0.004 (4)	−0.001 (4)
C4B	0.023 (4)	0.021 (4)	0.047 (5)	0.005 (3)	−0.002 (3)	0.004 (4)
C7B	0.018 (4)	0.043 (5)	0.049 (5)	0.002 (4)	−0.005 (4)	−0.013 (4)
C6B	0.026 (4)	0.022 (5)	0.066 (6)	0.003 (3)	0.006 (4)	0.001 (4)
C8A	0.022 (4)	0.036 (5)	0.033 (4)	0.002 (3)	−0.001 (3)	0.009 (4)
C12A	0.051 (5)	0.041 (5)	0.074 (6)	0.014 (4)	0.018 (5)	−0.003 (5)
C3B	0.036 (5)	0.031 (5)	0.041 (5)	−0.003 (4)	0.002 (4)	0.002 (4)
C8B	0.035 (4)	0.030 (5)	0.035 (5)	0.004 (4)	−0.007 (4)	0.005 (4)
C2A	0.077 (7)	0.085 (7)	0.031 (5)	−0.010 (6)	−0.016 (5)	0.022 (5)
C11A	0.047 (5)	0.040 (5)	0.086 (7)	0.014 (4)	−0.014 (5)	0.003 (5)
C1A	0.059 (11)	0.052 (11)	0.110 (10)	0.007 (9)	0.001 (9)	−0.008 (9)
C11B	0.078 (7)	0.081 (8)	0.064 (7)	0.022 (6)	−0.005 (5)	−0.003 (6)
C2B	0.040 (5)	0.035 (5)	0.086 (7)	−0.008 (4)	−0.011 (5)	−0.021 (5)
C12B	0.105 (9)	0.102 (9)	0.068 (7)	0.002 (7)	0.030 (6)	−0.015 (7)
C1B	0.058 (6)	0.048 (6)	0.090 (7)	−0.001 (5)	0.021 (5)	0.001 (6)

### Geometric parameters (Å, °)

**Table:**

I1B—C9B	2.119 (8)	C9A—C4A	1.403 (10)
I1A—C9A	2.115 (8)	C9A—C8A	1.433 (11)
I3A—C7A	2.156 (8)	N1A—H1A1	0.8600
I2B—C5B	2.171 (8)	N1A—H1A2	0.8600
I3B—C7B	2.098 (8)	C10A—C8A	1.515 (11)
I2A—C5A	2.107 (8)	C4B—C3B	1.517 (11)
C7A—C8A	1.370 (12)	C7B—C8B	1.386 (12)
C7A—C6A	1.418 (11)	C7B—C6B	1.421 (13)
O1A—C3A	1.334 (11)	C12A—C11A	1.477 (12)
O1A—C2A	1.495 (4)	C12A—H12A	0.9600
O3B—C10B	1.333 (9)	C12A—H12B	0.9600
O3B—C11B	1.496 (4)	C12A—H12C	0.9600
O1B—C3B	1.347 (9)	C2A—C1A	1.521 (12)
O1B—C2B	1.496 (6)	C2A—H2A1	0.9700
O4A—C10A	1.182 (9)	C2A—H2A2	0.9700
O3A—C10A	1.321 (9)	C11A—H11A	0.9700
O3A—C11A	1.495 (9)	C11A—H11B	0.9700
C3A—O2A	1.186 (10)	C1A—H1A3	0.9600
C3A—C4A	1.521 (12)	C1A—H1A4	0.9600
O2B—C3B	1.197 (10)	C1A—H1A5	0.9600
O4B—C10B	1.191 (10)	C11B—C12B	1.521 (6)
C5B—C4B	1.397 (11)	C11B—H11C	0.9700
C5B—C6B	1.405 (12)	C11B—H11D	0.9700
C10B—C8B	1.547 (11)	C2B—C1B	1.522 (7)
C6A—N1A	1.384 (11)	C2B—H2B1	0.9700
C6A—C5A	1.432 (12)	C2B—H2B2	0.9700
C5A—C4A	1.431 (11)	C12B—H12D	0.9600
C9B—C4B	1.405 (11)	C12B—H12E	0.9600
C9B—C8B	1.412 (11)	C12B—H12F	0.9600
N1B—C6B	1.390 (11)	C1B—H1B3	0.9600
N1B—H1B1	0.8600	C1B—H1B4	0.9600
N1B—H1B2	0.8600	C1B—H1B5	0.9600
			
C8A—C7A—C6A	120.7 (7)	C11A—C12A—H12B	109.5
C8A—C7A—I3A	120.3 (6)	H12A—C12A—H12B	109.5
C6A—C7A—I3A	119.0 (6)	C11A—C12A—H12C	109.5
C3A—O1A—C2A	117.8 (6)	H12A—C12A—H12C	109.5
C10B—O3B—C11B	116.3 (5)	H12B—C12A—H12C	109.5
C3B—O1B—C2B	115.8 (5)	O2B—C3B—O1B	125.2 (8)
C10A—O3A—C11A	117.6 (5)	O2B—C3B—C4B	125.1 (8)
O2A—C3A—O1A	123.3 (8)	O1B—C3B—C4B	109.7 (6)
O2A—C3A—C4A	124.3 (9)	C7B—C8B—C9B	119.5 (7)
O1A—C3A—C4A	112.4 (7)	C7B—C8B—C10B	120.9 (7)
C4B—C5B—C6B	120.3 (8)	C9B—C8B—C10B	119.5 (7)
C4B—C5B—I2B	119.2 (6)	O1A—C2A—C1A	115.0 (7)
C6B—C5B—I2B	120.4 (6)	O1A—C2A—H2A1	108.5
O4B—C10B—O3B	123.3 (7)	C1A—C2A—H2A1	108.5
O4B—C10B—C8B	125.3 (8)	O1A—C2A—H2A2	108.5
O3B—C10B—C8B	111.4 (6)	C1A—C2A—H2A2	108.5
N1A—C6A—C7A	121.6 (8)	H2A1—C2A—H2A2	107.5
N1A—C6A—C5A	120.9 (8)	C12A—C11A—O3A	104.8 (6)
C7A—C6A—C5A	117.4 (7)	C12A—C11A—H11A	110.8
C4A—C5A—C6A	122.7 (7)	O3A—C11A—H11A	110.8
C4A—C5A—I2A	116.6 (6)	C12A—C11A—H11B	110.8
C6A—C5A—I2A	120.7 (6)	O3A—C11A—H11B	110.8
C4B—C9B—C8B	119.7 (7)	H11A—C11A—H11B	108.9
C4B—C9B—I1B	121.0 (5)	C2A—C1A—H1A3	109.5
C8B—C9B—I1B	119.0 (6)	C2A—C1A—H1A4	109.5
C6B—N1B—H1B1	120.0	H1A3—C1A—H1A4	109.5
C6B—N1B—H1B2	120.0	C2A—C1A—H1A5	109.5
H1B1—N1B—H1B2	120.0	H1A3—C1A—H1A5	109.5
C4A—C9A—C8A	120.4 (7)	H1A4—C1A—H1A5	109.5
C4A—C9A—I1A	118.1 (6)	O3B—C11B—C12B	111.3 (4)
C8A—C9A—I1A	121.4 (5)	O3B—C11B—H11C	109.4
C6A—N1A—H1A1	120.0	C12B—C11B—H11C	109.4
C6A—N1A—H1A2	120.0	O3B—C11B—H11D	109.4
H1A1—N1A—H1A2	120.0	C12B—C11B—H11D	109.4
C9A—C4A—C5A	117.1 (7)	H11C—C11B—H11D	108.0
C9A—C4A—C3A	122.2 (7)	O1B—C2B—C1B	104.8 (4)
C5A—C4A—C3A	120.7 (7)	O1B—C2B—H2B1	110.8
O4A—C10A—O3A	124.5 (7)	C1B—C2B—H2B1	110.8
O4A—C10A—C8A	125.0 (8)	O1B—C2B—H2B2	110.8
O3A—C10A—C8A	110.5 (6)	C1B—C2B—H2B2	110.8
C5B—C4B—C9B	120.5 (7)	H2B1—C2B—H2B2	108.9
C5B—C4B—C3B	118.9 (7)	C11B—C12B—H12D	109.5
C9B—C4B—C3B	120.6 (7)	C11B—C12B—H12E	109.5
C8B—C7B—C6B	121.1 (7)	H12D—C12B—H12E	109.5
C8B—C7B—I3B	118.1 (6)	C11B—C12B—H12F	109.5
C6B—C7B—I3B	120.7 (6)	H12D—C12B—H12F	109.5
N1B—C6B—C5B	118.7 (8)	H12E—C12B—H12F	109.5
N1B—C6B—C7B	122.5 (8)	C2B—C1B—H1B3	109.5
C5B—C6B—C7B	118.7 (8)	C2B—C1B—H1B4	109.5
C7A—C8A—C9A	121.6 (7)	H1B3—C1B—H1B4	109.5
C7A—C8A—C10A	119.9 (7)	C2B—C1B—H1B5	109.5
C9A—C8A—C10A	118.6 (7)	H1B3—C1B—H1B5	109.5
C11A—C12A—H12A	109.5	H1B4—C1B—H1B5	109.5
			
C2A—O1A—C3A—O2A	4.7 (12)	C8B—C7B—C6B—N1B	−178.5 (8)
C2A—O1A—C3A—C4A	−175.0 (6)	I3B—C7B—C6B—N1B	−1.7 (11)
C11B—O3B—C10B—O4B	−0.5 (12)	C8B—C7B—C6B—C5B	6.0 (12)
C11B—O3B—C10B—C8B	176.6 (6)	I3B—C7B—C6B—C5B	−177.3 (6)
C8A—C7A—C6A—N1A	−179.1 (8)	C6A—C7A—C8A—C9A	−1.4 (12)
I3A—C7A—C6A—N1A	2.4 (11)	I3A—C7A—C8A—C9A	177.0 (6)
C8A—C7A—C6A—C5A	3.9 (12)	C6A—C7A—C8A—C10A	178.5 (7)
I3A—C7A—C6A—C5A	−174.6 (6)	I3A—C7A—C8A—C10A	−3.1 (10)
N1A—C6A—C5A—C4A	179.6 (8)	C4A—C9A—C8A—C7A	−1.8 (12)
C7A—C6A—C5A—C4A	−3.4 (12)	I1A—C9A—C8A—C7A	174.2 (6)
N1A—C6A—C5A—I2A	−0.1 (12)	C4A—C9A—C8A—C10A	178.3 (7)
C7A—C6A—C5A—I2A	176.9 (6)	I1A—C9A—C8A—C10A	−5.7 (10)
C8A—C9A—C4A—C5A	2.3 (11)	O4A—C10A—C8A—C7A	−72.2 (12)
I1A—C9A—C4A—C5A	−173.8 (6)	O3A—C10A—C8A—C7A	106.5 (9)
C8A—C9A—C4A—C3A	−178.2 (7)	O4A—C10A—C8A—C9A	107.7 (10)
I1A—C9A—C4A—C3A	5.6 (10)	O3A—C10A—C8A—C9A	−73.6 (9)
C6A—C5A—C4A—C9A	0.3 (12)	C2B—O1B—C3B—O2B	8.9 (12)
I2A—C5A—C4A—C9A	180.0 (6)	C2B—O1B—C3B—C4B	−169.2 (6)
C6A—C5A—C4A—C3A	−179.2 (8)	C5B—C4B—C3B—O2B	73.7 (12)
I2A—C5A—C4A—C3A	0.5 (10)	C9B—C4B—C3B—O2B	−104.8 (10)
O2A—C3A—C4A—C9A	89.2 (11)	C5B—C4B—C3B—O1B	−108.2 (8)
O1A—C3A—C4A—C9A	−91.1 (9)	C9B—C4B—C3B—O1B	73.3 (10)
O2A—C3A—C4A—C5A	−91.4 (11)	C6B—C7B—C8B—C9B	−4.6 (12)
O1A—C3A—C4A—C5A	88.3 (9)	I3B—C7B—C8B—C9B	178.6 (6)
C11A—O3A—C10A—O4A	−9.2 (13)	C6B—C7B—C8B—C10B	178.4 (7)
C11A—O3A—C10A—C8A	172.1 (7)	I3B—C7B—C8B—C10B	1.6 (10)
C6B—C5B—C4B—C9B	0.1 (12)	C4B—C9B—C8B—C7B	0.9 (12)
I2B—C5B—C4B—C9B	−176.5 (6)	I1B—C9B—C8B—C7B	176.3 (6)
C6B—C5B—C4B—C3B	−178.4 (7)	C4B—C9B—C8B—C10B	178.0 (7)
I2B—C5B—C4B—C3B	5.0 (10)	I1B—C9B—C8B—C10B	−6.7 (10)
C8B—C9B—C4B—C5B	1.4 (12)	O4B—C10B—C8B—C7B	89.1 (11)
I1B—C9B—C4B—C5B	−173.9 (6)	O3B—C10B—C8B—C7B	−87.9 (9)
C8B—C9B—C4B—C3B	179.8 (7)	O4B—C10B—C8B—C9B	−87.9 (11)
I1B—C9B—C4B—C3B	4.5 (10)	O3B—C10B—C8B—C9B	95.1 (9)
C4B—C5B—C6B—N1B	−179.4 (8)	C3A—O1A—C2A—C1A	−90.6 (9)
I2B—C5B—C6B—N1B	−2.9 (11)	C10A—O3A—C11A—C12A	−158.0 (8)
C4B—C5B—C6B—C7B	−3.6 (12)	C10B—O3B—C11B—C12B	177.5 (6)
I2B—C5B—C6B—C7B	172.9 (6)	C3B—O1B—C2B—C1B	157.5 (8)

### Hydrogen-bond geometry (Å, °)

**Table:**

*D*—H···*A*	*D*—H	H···*A*	*D*···*A*	*D*—H···*A*
C2A—H2A2···O2A^i^	0.97	2.54	3.411 (8)	150
C12A—H12A···O2B^ii^	0.96	2.60	3.545 (11)	169

Symmetry codes: (i) −*x*, −*y*+1, −*z*; (ii) *x*−1, *y*, *z*.
